# Decision in a Suit of Malpractice

**Published:** 1873-04

**Authors:** 


ART. V.—*Decision in a Suit of Malpractice.* State of New York. Court of Appeals. Carpenter vs. Blake, Dec. 1872. ALLEN, Judge.

It was a manifest error and prejudicial to the defendant, to charge that it was immaterial whether the defendant was, or was not reputed to be, or was, or was not, a skillful Surgeon. This was one of the material issues in the Case. If he had not competent skill, he was censurable for holding himself out to the public as possessing that skill, and the jury would properly hold him strictly accountable for the consequences of his acts.

He would not be entitled to the benefits, which would enure to the skillful Surgeon from an error of judgment, or mistake in the appliances and means at command of the expert. Not having skill or experience he could have no reasons, and could exercise no discretion, or choose between different methods, and having adopted a process, which was not successful, the exclusion of one which might, or probably would have proved successful, he cannot fall back for protection upon that which will serve as a shield to the skilled operator, that having skill and knowledge to choose between two or more courses of treatment, in the exercise of his judgment and with knowledge of all, he honestly and fairly chose the one to the exclusion of the other—*Leighton* vs. *Sargent 7th Foster*, *N. H.* 460 is in point. In that case a new trial was granted for the exclusion of evidence that the defendant was a skillful and experienced Surgeon. The learned Judge in a well reasoned opinion, reviews the cases and principles bearing upon the liability of professional men and experts for negligence and want of skill, and declares the general proposition, that one who offers himself for employment in a professional capacity undertakes,

1st. That he possesses that reasonable degree of learning and skill which is ordinarily possessed by the professors of the same art or science, and which is ordinarily regarded by the community and by those conversant with the employment as necessary to qualify him to engage in such business.

2nd. That he will use reasonable and ordinary care and diligence in the execution of his skill and the application of his knowledge, to accomplish the purpose for which it is employed.

3d. To use his best judgment in the exertion of his skill and the application of his diligence. The first and primary element of his engagement has respect to the possession of the ordinary and reasonable skill and learning necessary to qualify him for the service undertaken and as it was error in *Leighton* vs. *Sargent* to exclude evidence of the qualification, because it was material to the issue, it was error here to charge that the fact although proved was immaterial. It diverted the minds of the jury from one of the principal issues and from a fact if established, tending greatly to exonerate the defendant and shield him from liability. The Judge erred also in refusing to charge that the defendant had a right to withdraw from the case upon reasonable notice and a refusal so to charge, tended to mislead the jury, and give an impression that a surgeon withdrawing from a case, without the assent of the patient, given with full and accurate knowledge of his condition, would be liable for all the consequences that should result from the injury, thereafter. It was necessarily implied in the request that the surgeon did not leave in an energency when immediate action was to be had, or operation performed, or when other competent aid could not be had. The reasonable notice to be given qualified the right of the surgeon to withdraw as propounded in the request. A surgeon is not bound, in the absence of a special contract, to attend upon his patient any longer than the necessities of the case require, and having performed an operation to meet an exigency he can then withdraw, having the patient to secure other medical attendants to complete or watch over the case, and in such case the withdrawing surgeon is only liable for neglect or want of skill up to the time he commits the patient to other hands. Again the Judge erred in declining to charge that if the negligence of the plaintiff contributed to the injury complained of the defendant was not responsible. There was some evidence tending to show that the plaintiff by her own acts caused a reluxation of the arm, the defendant should not have been charged. The effect of the refusal of the Judge to charge as requested, was to withdraw this evidence from the consideration of the jury. Indeed, the reason assigned was that there was no evidence for submission to the jury on this branch of the case.

There were other statements of the charge open to criticism, and the whole case was one of great doubt and certainly the evidence to charge the defendant was not very conclusive. But perhaps there was evidence sufficient to carry the case to the jury. But if the rule contended for in some cases, to wit, that there must be manifest fault or gross negligence to charge a professional man as for a negligent or unskillful performance of duty, that is *lata culpa vel crassa negligentia*, it would not be a case for a jury. *Godfrey* vs. *Dalton*, 6 *Ring.* 460. *Wilson* vs. *Russ*, 7 *Shepley* 421. But without considering further the line which divides the reasonable skill and diligence which satisfies the undertaking of the surgeon, or attorney, and that gross negligence or palpable fault for which they are responsible, for the errors in the charge suggested, the judgment must be reversed and a new trial granted.

"Peckham J. reads for affirmance. Grover J. and chief concur. Allen J. reads for reversal and new trial. Folger, Rapallo and Andrews J. s. concur."

NOTE.—Though the point was not before the Court, there is a strong intimation that if it had been, the Court would have decided that a nonsuit should have been granted, as contended for in my paper, published in *Buffalo Medical Journal*, July 1872.

H. F. MONTGOMERY, M. D.

